# Diffuse Cutaneous Metastases as the Only Sign of Extranodal Tumor Spread in a Patient with Adenocarcinoma of the Colon

**DOI:** 10.5402/2011/902971

**Published:** 2011-06-14

**Authors:** Serenella Civitelli, Barbara Civitelli, Jacopo Martellucci, Gabriello Tanzini

**Affiliations:** Department of Surgery, University of Siena, Policlinico le Scotte, Viale Bracci, 53100 Siena, Italy

## Abstract

Cutaneous metastases from large bowel cancer are uncommon and are usually associated with organ involvement. Localization of lesions to the skin is mainly attributed to vascular and anatomical relationship, since most of them are seen in the abdominal wall or in a surgical scar. We report a 73-year-old woman in whom metastatic nodules from a poorly differentiated adenocarcinoma of the right colon developed throughout the skin (buttock, trunk, chest wall, arms, and neck) and remained the only sign of extranodal tumor spread until patient's death, seven months later. This unusual behaviour suggests that localization of neoplastic cells to the skin may be a site-specific process, determined by adhesion molecules and/or by growth factors found at that site.

## 1. Introduction

Cutaneous metastases from large bowel cancer are uncommon, occurring in less than 4% of patients [1, 2]. Localization of lesions is mainly attributed to vascular and anatomical relationship since most of them are seen in the skin of the abdominal wall or in a surgical scar [1, 3, 4]. Distant lesions are generally associated with organ involvement [5–7]. The case we report is unusual because multiple metastatic nodules appeared after removal of an adenocarcinoma of the right colon and developed throughout the skin (buttock, trunk, chest wall, arms, and neck) as the only sign of extranodal tumor spread until patient's death, seven months later.

## 2. Case Report

**Figure 1:**
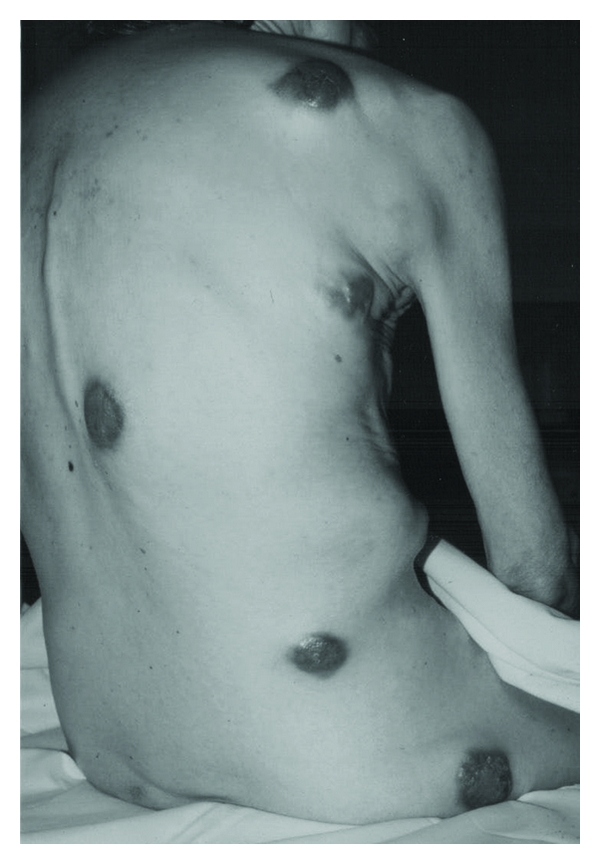
Cutaneous metastasis.

A 73-year-old woman was admitted to our hospital with a few days history of fever and diarrhoea. She reported a 3-month weight loss of about 4 kg and complained of a mild abdominal pain in the right iliac fossa. A colonoscopy revealed an adenocarcinoma of the ascending colon. Chest X-ray and abdominal ultrasonography showed no pulmonary or liver metastases. A right hemicolectomy was performed. Histological examination of surgical specimen revealed a poorly differentiated adenocarcinoma, 4  ×  4 cm in diameter, with invasion of the serosa and metastases to mesenteric lymph nodes. A few days after discharge, the patient noted three nodules in her abdominal wall, chest, and back skin respectively. On physical examination, the nodules were indolent, elastic masses of about 2  ×  2 cm in diameter, not freely movable, with normal appearance of the overlying skin. Excisional biopsy revealed cutaneous metastases from adenocarcinoma. The patient refused any adjuvant treatment. Six months later, she was readmitted for removal of a large, firm, flesh, coloured mass of her left arm, limiting the movements. By that moment, multiple nodules were present throughout the skin of the buttock, trunk, abdominal wall, and neck (Figure 1). 

The lesions were varying in size from few millimeters to 10 centimetres. The overlying skin was normal or violaceous. A nodule on the chest wall was ulcerated. Chest X-ray and bone scintigraphy showed no metastatic lesions. Abdominal ultrasonography revealed no sign of abdominal relapse, in particular no liver secondaries. In the following days, the patient developed a rapidly increasing mild dyspnoea. Indirect laryngoscopy showed incomplete paralysis of the vocal cords. A tracheostomy was performed as an emergency procedure for an asphyxial crisis. On gross appearance, the trachea was compressed and infiltrated by large subcutaneous masses. Results of histological examination revealed metastases from adenocarcinoma. The patient died of respiratory failure before any other therapeutic options could be undertaken. Autopsy was not performed. 

## 3. Discussion

Cutaneous metastases are rare. Their incidence report ranges from 0,7 to 10,5% in autoptic and retrospective studies [4, 7]. In order of decreasing frequency, the sources are breast and lung cancer, melanoma, and squamous carcinoma of the upper tract. Among gastrointestinal malignancies, gastric cancer is the most common neoplasm to present with skin involvement [2–8]. Cutaneous metastases from large bowel carcinoma account for approximately 5% of all skin secondaries [1, 2]. Liver, peritoneal, and lung involvement is generally associated [3, 4, 8, 9]. The gross appearance of lesions is not distinctive, and biopsy is often needed for the diagnosis. Usually they appear as painless nodules within the dermis and subcutaneous tissue, with intact and uninvolved epidermis, within the first two years after resection of the primary tumor [1, 3, 5]. Cutaneous metastases may be the first clinical sign of relapse or, more rarely, may reveal an asymptomatic malignancy [4]. Anyway, they generally represent a poor prognostic sign. Several explanations for their development have been proposed. Direct extension through lymphatic or surgical tracts seems to be an important mechanism since most of lesions are located in the skin overlying the abdominal wall, in a colostomy site or in the surgical incision [1, 3, 4]. Metastases at the trocar site or at the minilaparotomy incision have been reported after laparoscopic-assisted colectomy [10]. Implantation during surgery may contribute to this type of recurrence. Other possible routes of dissemination are extensions along ligaments of common embryonic origin, such as the round ligament of the liver. Metastasis to the umbilicus from an internal malignancy is well known as the “Sister Mary Joseph's nodule” [11]. Distant metastases, in order of decreasing frequency, have been seen in pelvis, upper extremities, chest, and back skin. There are rare reports of metastases of the head, neck, tongue, lip, and hands [4, 8, 12–14]. Remote lesions are usually associated with organ involvement and are believed to be due to diffuse hematogenous dissemination of tumor cells and to their trapping in the capillary bed of the skin, just for mechanical factors. 

The case we report is unusual because remote cutaneous metastases are not associated with visceral secondaries. It seems unlikely that the tumor cells locate throughout the skin just for mechanical factors without hepatic or pulmonary involvement, since lung and liver receive all of the venous drainage from the colon, prior to distribution to the rest of the body. It seems reasonable to suppose that circulating tumor cells bind specifically to the skin by site-specific adhesion molecules and/or respond preferentially to growth factors found at that site.
